# Supplementary material for: RNA-Seq Time Series of Vitis vinifera Bud Development Reveals Correlation of Expression Patterns with the Local Temperature Profile
Source: Plants (Basel). 2020 Nov 12;9(11):1548. doi: 10.3390/plants9111548 (PMC7698159; doi:10.3390/plants9111548)
Supplement: Supplementary file 1 [file plants-09-01548-s001.zip › Supplement-files-final_proof/File-S6_heatmap-MADS-box-genes.pdf]

	2016-06-01	2016-06-02	2016-06-04	2016-06-06	2016-06-09	2016-06-12	2016-06-14	2016-06-16	2016-06-18	2016-06-21	2016-06-24	2016-06-28	2016-07-26	2016-08-04	2016-08-11	2016-08-23	2016-09-08	2016-09-22	2016-11-03
VIT_200s0211g00140-VviAGL17d	2	1	2	2	2	2	2	3	2	2	3	3	9	4	8	12	16	11	10
VIT_200s0211g00180-VviAGL17c	1	1	1	1	1	1	1	2	1	1	2	1	5	3	4	5	8	5	4
VIT_200s0313g00070-VviSVP1	33	34	38	37	28	36	34	38	35	33	36	31	40	33	42	33	43	48	31
VIT_201s0011g00100-VviAP1	0	0	0	0	0	1	0	1	0	0	1	1	11	4	13	7	12	18	11
VIT_201s0011g00110-VviSEP4	1	1	0	1	1	1	2	1	1	2	2	2	1	1	1	1	1	1	0
VIT_202s0025g04650-VviSOC1c	27	28	27	20	25	24	23	23	24	19	18	22	12	13	14	10	8	10	12
VIT_203s0017g00360-VviSVP4	11	9	10	11	9	9	7	7	8	7	9	7	5	4	5	4	3	3	1
VIT_203s0017g00440-VviSVPS1	21	19	24	23	18	21	16	20	18	18	22	18	17	15	16	21	18	11	11
VIT_203s0097g00160-VviAGL17b	3	3	3	3	3	2	4	2	2	3	1	3	5	4	5	6	9	8	6
VIT_203s0167g00070-VviSVP5	14	15	17	16	14	14	15	17	16	13	18	14	21	17	22	22	31	19	14
VIT_204s0008g01980-VviMADSD2a	3	2	4	5	4	3	3	2	4	2	4	1	0	1	0	0	0	0	0
VIT_214s0068g01800-VviFLC2	14	14	18	12	10	13	14	20	13	13	9	14	43	41	46	53	57	116	71
VIT_214s0083g01030-VviFUL2	0	0	0	1	0	1	1	0	0	1	1	1	2	1	2	1	2	2	2
VIT_215s0024g01860-VviSVPS4	3	3	3	3	2	3	4	3	3	2	3	2	2	1	2	2	2	1	1
VIT_215s0048g01250-VviSOC1a	53	49	66	48	45	45	42	49	45	42	47	42	37	47	43	27	28	33	12
VIT_215s0107g00120-VviSVP3	16	14	21	21	13	14	16	15	15	12	18	12	19	14	17	18	28	13	12
VIT_216s0022g02330-VviAGL6b	0	1	0	0	0	0	1	0	0	0	0	1	4	1	3	3	4	4	3
VIT_216s0022g02380-VviSOC1b	18	18	19	17	13	16	16	17	15	15	17	13	18	21	20	15	20	25	13
VIT_217s0000g01230-VviTM8a	19	24	16	21	39	40	38	40	31	58	55	69	18	34	10	25	11	2	5
VIT_217s0000g04990-VviFUL1	20	21	19	17	20	23	31	24	24	27	28	28	22	26	25	20	14	17	21
VIT_217s0000g06340-VviMADSD2b	9	10	11	10	9	10	11	14	11	10	13	11	20	16	17	25	26	17	22
VIT_218s0001g07460-VviSVP2	24	20	25	25	22	19	20	15	18	18	20	16	21	21	19	22	18	17	17
VIT_218s0001g07900-VviAGL17a	3	2	3	3	3	3	3	2	2	3	3	3	4	4	4	6	10	6	4
VIT_218s0001g09540-VviMADSD1c	0	0	0	0	0	0	0	0	0	0	0	0	1	0	1	1	1	2	10
VIT_218s0001g13460-VviAP3a	1	1	1	1	1	1	1	1	1	1	1	1	1	1	1	1	2	2	0
VIT_218s0041g02140-VviAGL12	3	2	3	2	2	2	2	3	3	3	3	2	5	5	4	4	3	1	0
VIT_230s0000g00009-VviFLC1	6	6	7	7	4	6	6	8	6	6	7	9	7	5	7	10	7	12	24
VIT_230s0000g00011-VviMADS1A2d	6	6	4	5	6	5	4	3	6	6	4	4	0	2	1	1	0	0	0
