# Supplementary material for: RNA-Seq Time Series of Vitis vinifera Bud Development Reveals Correlation of Expression Patterns with the Local Temperature Profile
Source: Plants (Basel). 2020 Nov 12;9(11):1548. doi: 10.3390/plants9111548 (PMC7698159; doi:10.3390/plants9111548)
Supplement: Supplementary file 1 [file plants-09-01548-s001.zip › Supplement-files-final_proof/File-S8_heatmap-WRKY-genes.pdf]

	2016-06-01	2016-06-02	2016-06-04	2016-06-06	2016-06-09	2016-06-12	2016-06-14	2016-06-16	2016-06-18	2016-06-21	2016-06-24	2016-06-28	2016-07-26	2016-08-04	2016-08-11	2016-08-23	2016-09-08	2016-09-22	2016-11-03
VIT_201s0011g00720-VviWRKY01	10	12	12	14	11	14	14	20	16	17	15	17	21	25	21	16	14	15	6
VIT_201s0010g03930-VviWRKY03	2	2	2	2	3	3	2	2	2	3	2	2	16	15	11	11	16	15	25
VIT_204s0008g05760-VviWRKY08	25	23	67	34	22	20	20	20	23	29	30	29	25	35	31	23	31	18	46
VIT_204s0008g06600-VviWRKY09	8	7	6	6	6	6	7	4	5	6	6	5	4	4	4	4	4	4	8
VIT_204s0069g00920-VviWRKY10	15	20	26	18	17	22	18	22	20	22	24	22	31	37	31	28	27	21	29
VIT_204s0023g00470-VviWRKY13	2	2	2	2	1	2	2	2	2	2	2	2	3	2	3	2	2	2	1
VIT_205s0077g00730-VviWRKY14	3	3	3	3	2	4	4	5	3	4	4	5	13	17	15	10	8	6	14
VIT_206s0004g00230-VviWRKY15	14	11	25	13	8	8	12	13	12	13	12	12	7	8	7	9	14	8	2
VIT_206s0004g07500-VviWRKY16	33	31	36	18	25	31	41	42	27	33	23	41	81	89	94	76	80	93	332
VIT_207s0141g00680-VviWRKY17	19	21	22	22	20	22	19	20	19	22	23	22	23	22	24	19	19	18	10
VIT_207s0005g01520-VviWRKY18	11	16	22	18	13	18	22	26	15	19	22	25	50	65	44	46	49	41	18
VIT_207s0005g01710-VviWRKY19	12	17	15	15	16	20	29	24	20	23	21	25	45	44	32	46	34	44	29
VIT_207s0005g02570-VviWRKY20	13	13	17	11	13	18	27	27	14	20	14	26	79	100	87	44	39	29	26
VIT_207s0031g00080-VviWRKY21	16	18	21	17	16	21	18	20	18	20	19	21	27	31	25	21	21	16	10
VIT_207s0031g01710-VviWRKY22	10	8	15	8	8	9	9	8	8	9	9	8	3	7	3	2	4	2	0
VIT_207s0031g01840-VviWRKY23	39	58	41	42	65	66	82	69	49	84	56	76	23	29	21	27	11	7	9
VIT_208s0058g01390-VviWRKY25	81	89	202	124	91	87	113	120	104	131	123	131	97	148	125	95	114	49	4
VIT_208s0040g03070-VviWRKY26	18	20	20	19	16	19	20	24	20	18	20	20	25	24	27	23	28	22	17
VIT_208s0007g00570-VviWRKY27	0	1	1	1	1	1	1	1	1	1	1	1	5	6	4	4	4	4	3
VIT_209s0018g00240-VviWRKY28	3	2	4	1	2	3	8	2	2	3	1	3	3	4	3	4	4	4	9
VIT_210s0116g01200-VviWRKY29	7	4	6	3	4	5	6	5	4	6	5	6	14	18	14	14	15	18	40
VIT_210s0003g01600-VviWRKY30	6	7	9	6	6	8	8	12	8	10	6	12	29	43	32	26	23	24	36
VIT_210s0003g02810-VviWRKY31	4	6	8	4	3	6	4	8	6	6	4	6	29	30	30	20	13	14	26
VIT_210s0003g05740-VviWRKY32	5	5	5	7	5	6	5	5	6	6	8	6	5	6	5	5	3	3	9
VIT_211s0037g00150-VviWRKY33	16	19	17	14	15	19	21	23	18	19	20	22	29	28	27	35	39	32	61
VIT_211s0052g00450-VviWRKY34	16	16	22	17	18	21	18	17	18	19	21	17	18	21	19	16	14	12	7
VIT_212s0028g00270-VviWRKY35	3	4	5	3	2	5	4	5	5	5	5	5	25	26	25	23	23	17	15
VIT_212s0059g00880-VviWRKY37	5	5	7	4	4	4	6	6	5	7	4	6	6	10	8	5	5	5	14
VIT_212s0057g00550-VviWRKY38	10	10	9	8	9	10	10	10	10	9	9	10	13	13	14	13	12	14	17
VIT_212s0055g00340-VviWRKY39	0	0	0	0	0	0	0	1	0	0	0	0	4	4	3	4	4	6	18
VIT_213s0067g03140-VviWRKY41	25	25	35	27	20	24	42	21	24	31	20	26	7	13	11	5	6	3	1
VIT_214s0081g00560-VviWRKY42	15	18	16	14	15	17	20	16	14	17	19	22	24	24	20	22	22	18	14
VIT_214s0108g01280-VviWRKY45	51	39	42	30	36	43	61	41	35	43	33	45	60	70	64	62	55	52	131
VIT_215s0021g01310-VviWRKY46	15	18	19	17	18	17	15	19	18	15	17	16	9	8	9	15	6	6	10
VIT_215s0046g01140-VviWRKY47	7	6	12	7	6	8	9	6	6	7	6	7	5	8	6	4	5	3	1
VIT_215s0046g02150-VviWRKY48	0	0	0	1	0	1	1	1	1	2	2	2	19	36	20	33	30	35	17
VIT_215s0046g02190-VviWRKY49	3	4	3	3	3	3	3	3	3	3	3	4	7	9	6	8	8	10	10
VIT_217s0000g01280-VviWRKY52	4	3	4	2	3	3	5	5	3	5	3	4	14	20	19	12	6	6	16
VIT_218s0001g10030-VviWRKY54	3	4	5	4	4	5	4	4	4	5	5	4	10	11	9	7	6	3	5
VIT_219s0090g00840-VviWRKY55	2	1	2	1	1	2	3	3	1	3	2	3	37	37	33	22	19	10	14
VIT_219s0090g01720-VviWRKY56	7	7	7	5	6	7	8	8	7	7	6	7	7	8	9	7	7	9	6
VIT_219s0015g01870-VviWRKY57	9	9	9	9	9	9	9	10	10	10	10	10	11	12	11	10	10	9	10
VIT_201s0011g00220-VviWRKY58	10	10	12	9	8	10	11	11	10	10	10	11	11	14	14	8	9	9	9
VIT_200s0463g00010-VviWRKY59	6	7	9	7	6	7	8	9	7	7	7	7	10	8	10	9	8	6	6
