# Supplementary material for: RNA-Seq Time Series of Vitis vinifera Bud Development Reveals Correlation of Expression Patterns with the Local Temperature Profile
Source: Plants (Basel). 2020 Nov 12;9(11):1548. doi: 10.3390/plants9111548 (PMC7698159; doi:10.3390/plants9111548)
Supplement: Supplementary file 1 [file File-S7_heatmap-R2R3-MYB-genes.pdf › Supplement-files-final_proof/File-S7_heatmap-R2R3-MYB-genes.pdf]

	2016-06-01	2016-06-02	2016-06-04	2016-06-06	2016-06-09	2016-06-12	2016-06-14	2016-06-16	2016-06-18	2016-06-21	2016-06-24	2016-06-28	2016-07-26	2016-08-04	2016-08-11	2016-08-23	2016-09-08	2016-09-22	2016-11-03
VIT_200s0203g00070-MYB143	2	2	3	1	1	3	3	3	2	3	2	4	15	16	16	16	15	14	7
VIT_200s0203g00170-MYB136	4	5	12	11	6	6	5	8	6	7	10	7	7	9	6	4	6	2	0
VIT_200s0341g00050-MYBPAL1	6	6	6	8	6	7	6	7	7	7	7	7	7	8	8	6	7	10	9
VIT_200s1241g00010-MYB190	1	1	1	2	3	4	5	14	4	9	10	15	17	17	17	28	19	17	7
VIT_200s1352g00010-MYB148	1	1	1	1	1	1	1	1	1	1	1	1	6	8	5	6	3	5	4
VIT_201s0011g04760-MYBC2-L1	15	24	25	28	19	18	13	30	29	19	23	20	43	51	49	26	26	26	5
VIT_201s0026g01910-MYB192	24	20	21	21	20	23	26	20	21	24	29	28	20	21	19	21	13	9	2
VIT_201s0026g02600-MYB165	10	10	9	10	8	9	13	12	9	8	8	9	12	9	9	6	11	8	2
VIT_201s0026g02770-MYB140	25	32	33	34	25	36	47	59	43	42	47	42	35	38	25	11	40	9	0
VIT_202s0012g01650-MYB178	0	0	0	0	0	0	0	0	0	0	0	0	4	5	4	4	3	4	1
VIT_202s0025g00320-MYB103	2	2	2	3	3	3	3	11	4	9	12	11	4	4	4	5	2	1	0
VIT_203s0038g02310-MYB4A	22	23	28	28	29	29	37	33	29	39	34	41	59	73	65	54	40	29	17
VIT_203s0180g00210-MYB171	2	2	2	2	2	2	3	3	2	3	3	3	4	4	4	5	5	7	24
VIT_204s0008g00620-MYB153	3	5	6	7	5	6	4	8	7	7	8	6	15	15	15	9	9	6	0
VIT_204s0008g01800-MYBPA7	24	25	35	32	23	34	40	48	41	37	38	34	50	65	47	33	41	34	9
VIT_204s0008g01810-MYBPA8	9	10	7	9	10	8	4	7	11	5	6	4	2	3	3	2	2	3	0
VIT_204s0008g01820-MYBPA9	2	2	2	1	1	2	2	2	2	2	1	1	1	1	1	1	1	2	0
VIT_204s0023g03710-MYB4B	11	12	19	23	17	9	5	9	12	11	20	12	9	11	8	7	7	7	3
VIT_204s0044g01380-MYB166	0	0	0	0	0	0	0	1	0	1	1	1	2	2	1	3	2	2	5
VIT_205s0020g01100-MYB146	3	2	3	2	2	3	3	5	3	2	3	3	11	9	13	23	21	42	34
VIT_205s0049g01020-MYB15	2	2	3	1	2	2	2	3	1	2	1	2	4	5	4	4	5	8	139
VIT_205s0077g00500-MYB108A	4	4	10	6	5	4	5	7	5	6	7	6	11	14	11	11	9	8	22
VIT_206s0004g00570-MYB5B	17	18	18	18	16	16	17	24	22	18	17	18	25	29	30	25	28	43	18
VIT_206s0004g02110-MYB185	3	4	4	11	11	11	13	24	13	26	30	28	10	11	10	15	5	3	0
VIT_206s0004g04140-MYB187	3	4	2	2	2	4	6	2	3	3	5	7	14	5	5	8	5	4	7
VIT_206s0009g02480-MYB159	1	1	2	1	1	1	1	2	1	1	1	1	2	1	2	1	2	2	3
VIT_206s0061g00470-MYB149	12	13	15	12	11	15	18	23	15	16	15	17	35	37	36	33	39	36	8
VIT_207s0005g01210-MYBF1	13	9	9	10	9	9	9	11	11	7	7	7	5	5	5	3	7	7	2
VIT_207s0005g01950-MYB108B	1	0	0	0	0	0	0	1	1	0	0	0	2	1	2	3	1	1	7
VIT_207s0005g02480-MYB55A	16	13	15	12	15	15	25	13	13	19	16	19	12	13	13	11	8	9	2
VIT_207s0005g03340-MYB14	1	1	1	1	1	1	1	0	1	1	1	1	9	7	5	10	15	20	25
VIT_207s0130g00040-MYB3	16	18	20	20	17	23	26	33	24	23	25	27	39	48	40	35	50	65	40
VIT_207s0141g00100-MYB179	0	0	0	0	0	0	0	0	0	0	0	0	8	11	7	10	11	13	1
VIT_208s0007g00410-MYB91A	27	36	33	47	28	38	32	49	53	37	45	35	55	40	49	47	66	63	14
VIT_208s0007g01540-MYB176	10	12	12	10	12	13	13	17	13	14	12	15	17	17	18	21	22	34	29
VIT_208s0007g05030-MYB150	3	4	6	3	3	4	5	7	4	5	3	4	11	17	18	7	6	5	3
VIT_208s0056g00800-MYB60	4	5	7	6	5	6	5	9	7	6	6	6	10	11	8	7	13	8	2
VIT_208s0056g01190-MYB167	3	3	3	3	3	3	4	4	3	3	3	4	3	3	3	4	3	6	30
VIT_209s0002g01380-MYBPAL3	6	6	6	6	4	6	6	6	6	5	6	3	5	7	7	4	5	7	1
VIT_209s0002g01400-MYBPA5	2	2	2	3	2	2	3	4	2	3	4	3	3	3	4	2	5	3	1
VIT_211s0016g01300-MYBPAR	18	21	25	24	17	26	20	34	31	19	28	19	49	38	39	85	112	91	41
VIT_211s0016g01310-MYBPA4	1	1	1	2	1	1	1	1	1	1	2	1	1	1	1	1	2	1	0
VIT_211s0016g01320-MYBPA2	10	11	10	14	9	11	8	9	13	8	13	5	9	9	7	11	4	5	0
VIT_211s0016g02780-MYB154	1	0	1	1	1	1	0	1	1	1	0	1	1	2	1	1	1	1	1
VIT_212s0134g00490-MYB138	2	1	3	3	2	2	2	2	2	3	3	3	2	3	3	2	2	1	0
VIT_213s0019g03200-MYB177	5	6	6	4	5	6	6	7	5	5	5	6	9	9	10	8	7	10	11
VIT_213s0067g01630-MYB161	8	7	8	7	6	7	7	7	7	7	7	6	10	9	10	11	12	11	16
VIT_214s0006g01290-MYBA6	30	20	19	19	14	15	12	11	14	8	13	7	9	12	12	2	3	3	0
VIT_214s0006g01340-MYBA5	8	6	7	5	4	4	2	1	3	1	3	1	1	1	1	0	0	0	0
VIT_214s0006g01620-MYBC2-L3	1	1	1	3	2	1	1	2	2	2	3	1	2	2	2	1	2	1	0
VIT_214s0036g00460-MYB135	4	4	3	3	2	2	2	2	3	4	1	3	3	2	3	9	5	10	9
VIT_214s0060g00240-MYB55B	5	6	6	7	9	9	12	6	6	10	10	8	1	2	1	1	0	0	0
VIT_214s0066g01220-MYB156	4	4	5	4	3	4	4	5	4	3	4	3	5	5	5	4	4	5	1
VIT_214s0083g00120-MYB91B	17	20	18	22	15	20	20	23	21	21	18	20	18	23	20	11	16	23	12
VIT_214s0108g00830-MYB30B	17	14	17	16	11	15	22	28	18	18	16	18	18	25	21	10	14	16	4
VIT_214s0108g01010-MYB168	1	1	1	2	1	1	1	2	1	1	1	1	2	2	2	1	3	3	0
VIT_214s0108g01080-MYB141	14	13	19	20	14	15	15	21	16	17	19	21	34	33	32	37	57	52	26
VIT_215s0046g00170-MYBPA1	64	91	112	136	80	88	67	98	109	91	171	90	139	163	96	101	282	162	10
VIT_215s0046g03190-MYB17A	1	1	1	1	1	1	1	2	1	1	2	2	4	2	4	3	6	5	2
VIT_216s0022g01210-MYB191	2	3	4	7	4	3	2	4	5	4	9	5	2	3	2	3	3	2	0
VIT_216s0039g01710-MYB181	0	0	0	0	0	1	0	0	0	1	1	1	9	14	10	13	14	13	6
VIT_216s0039g01920-MYB196	24	27	24	20	26	27	24	24	24	25	20	25	24	28	27	22	19	28	16
VIT_216s0050g00070-MYB17B	3	4	4	5	3	4	4	6	4	5	4	5	10	7	11	12	21	33	8
VIT_217s0000g01890-MYB169	0	0	0	1	1	1	1	3	1	3	3	4	6	8	7	10	7	7	3
VIT_217s0000g02650-MYBC2-L4	5	7	8	10	7	9	9	12	10	10	12	9	22	24	23	20	31	40	12
VIT_217s0000g02660-MYBC2-L2	14	18	17	21	14	17	14	19	18	15	18	13	17	19	22	18	28	30	5
VIT_217s0000g03560-MYB164	5	6	6	4	3	4	5	7	4	4	4	4	17	19	16	18	14	19	38
VIT_217s0000g06190-MYB30A	13	15	14	14	11	16	14	21	15	12	17	14	31	32	33	38	55	67	120
VIT_217s0000g06410-MYB142	1	1	1	2	1	1	1	1	1	1	2	1	2	1	1	0	1	0	0
VIT_217s0000g08480-MYB157	23	17	19	15	15	14	14	15	13	12	11	11	7	9	12	6	7	19	10
VIT_217s0000g09080-MYB147	12	11	8	9	13	13	17	22	13	19	16	23	7	8	9	13	7	15	11
VIT_218s0001g05670-MYB182	0	0	0	0	0	0	0	0	0	0	0	1	7	8	5	8	6	8	1
VIT_218s0001g08470-MYB152	0	0	1	0	0	1	1	1	0	1	1	1	9	10	7	10	6	6	2
VIT_218s0001g09850-MYB174	90	71	102	65	66	66	93	111	57	68	79	85	108	101	115	126	131	135	143
VIT_218s0001g11170-MYB175	6	4	6	8	4	4	8	7	4	5	7	6	10	12	10	14	15	24	8
VIT_218s0001g15260-MYB170	0	0	0	0	0	1	1	1	1	1	1	2	4	5	4	4	5	6	5
VIT_219s0014g03820-MYB145	4	6	8	5	4	9	7	9	6	9	8	10	56	63	59	33	42	27	9
VIT_219s0015g01280-MYB80	1	1	1	0	0	1	1	1	1	1	1	1	1	2	1	1	1	3	4
VIT_219s0085g00050-MYB139	1	1	2	1	1	2	1	2	2	2	2	2	15	13	14	14	15	18	15
VIT_219s0085g00940-MYB67B	3	3	4	5	2	3	3	3	3	4	4	3	10	17	9	10	12	11	3
